# Comparing the blood oxygen level–dependent fluctuation power of benign and malignant musculoskeletal tumors using functional magnetic resonance imaging

**DOI:** 10.3389/fonc.2022.794555

**Published:** 2022-08-12

**Authors:** Lisha Duan, Huiyuan Huang, Feng Sun, Zhenjiang Zhao, Mengjun Wang, Mei Xing, Yufeng Zang, Xiaofei Xiu, Meng Wang, Hong Yu, Jianling Cui, Han Zhang

**Affiliations:** ^1^ Department of Radiology, the Third Hospital of Hebei Medical University, Shijiazhuang, China; ^2^ Key Laboratory of Biomechanics of Hebei Province, Shijiazhuang, China; ^3^ Center for Cognition and Brain Disorders, Hangzhou Normal University, Hangzhou, China; ^4^ School of Public Health and Management, Guangzhou University of Chinese Medicine, Guangzhou, China; ^5^ Zhejiang Key Laboratory for Research in Assessment of Cognitive Impairments, Hangzhou, China; ^6^ Department of Pathology, The Third Hospital of Hebei Medical University, Shijiazhuang, China; ^7^ Department of Radiology, the First Hospital of Hebei Medical University, Shijiazhuang, China; ^8^ School of Biomedical Engineering, ShanghaiTech University, Shanghai, China

**Keywords:** blood oxygen level-dependent, musculoskeletal tumors, functional magnetic resonance imaging, benign, malignant

## Abstract

**Purpose:**

The aim of this study is to compare the blood oxygen level–dependent (BOLD) fluctuation power in 96 frequency points ranging from 0 to 0.25 Hz between benign and malignant musculoskeletal (MSK) tumors *via* power spectrum analyses using functional magnetic resonance imaging (fMRI).

**Materials and methods:**

BOLD-fMRI and T1-weighted imaging (T1WI) of 92 patients with benign or malignant MSK tumors were acquired by 1.5-T magnetic resonance scanner. For each patient, the tumor-related BOLD time series were extracted, and then, the power spectrum of BOLD time series was calculated and was then divided into 96 frequency points. A two-sample *t*-test was used to assess whether there was a significant difference in the powers (the “power” is the square of the BOLD fluctuation amplitude with arbitrary unit) of each frequency point between benign and malignant MSK tumors. The receiver operator characteristic (ROC) analysis was used to assess the diagnostic capability of distinguishing between benign and malignant MSK tumors.

**Results:**

The result of the two-sample *t*-test showed that there was significant difference in the power between benign and malignant MSK tumor at frequency points of 58 (0.1508 Hz, *P* = 0.036), 59 (0.1534 Hz, *P* = 0.032), and 95 (0.247 Hz, *P* = 0.014), respectively. The ROC analysis of mean power of three frequency points showed that the area of under curve is 0.706 (*P =* 0.009), and the cutoff value is 0.73130. If the power of the tumor greater than or equal to 0.73130 is considered the possibility of benign tumor, then the diagnostic sensitivity and specificity values are 83% and 59%, respectively. The *post hoc* analysis showed that the merged power of 0.1508 and 0.1534 Hz in benign MSK tumors was significantly higher than that in malignant ones (*P* = 0.014). The ROC analysis showed that, if the benign MSK tumor was diagnosed with the power greater than or equal to the cutoff value of 1.41241, then the sensitivity and specificity were 67% and 68%, respectively.

**Conclusion:**

The mean power of three frequency points at 0.1508, 0.1534, and 0.247 Hz may potentially be a biomarker to differentiate benign from malignant MSK tumors. By combining the power of 0.1508 and 0.1534 Hz, we could better detect the difference between benign and malignant MSK tumors with higher specificity.

## Introduction

Differentiating malignant from benign musculoskeletal (MSK) tumors prior to clinical treatment is especially essential, as it may assist in formulating an appropriate treatment strategy, such as chemotherapy, radiation therapy, or surgery. Imaging evaluation of MSK tumors often involves a combination of modalities, such as plain radiography (1–3), computed tomography (4, 5), magnetic resonance imaging (MRI) (6, 7), scintigraphy (8), or ultrasonography (9, 10), and some functional MRI techniques (11), like diffusion-weighted imaging (DWI) (12), perfusion-weighted imaging (PWI) (13), and magnetic resonance spectroscopy (MRS) (14). These examinations can distinguish between most benign and malignant MSK tumors, but it is still difficult to identify some bone tumors and many soft tissue tumors. Although new developed state-of-the-art methods such as DWI, PWI, and MRS can help to differentiate between benign and malignant tumors, their sensitivity and specificity are low when only one of these techniques is used (15–18). Even biopsy findings may be inconclusive (19, 20), resulting in a delay in diagnosis and curative therapy. Hence, it is still of great value to find a new imaging feature to identify the malignancy of MSK tumors.

The structure of malignant tumor vasculature that is different from the benign one is typically disorganized, with blood vessels that are tortuous, dilated, elongated and leaky and with saccular and dead-end formations (21–24). This kind of vascular architecture may contribute to the presence of regions of persistent low oxygen tension and the temporally variable oxygenation.

Many indexes are used to detect the pathophysiological status of blood flow, one of which is vasomotion (25, 26). Vasomotion is the oscillation of vascular tone, and caliber with frequency points in the range from 1 to 20 min(-1) is seen in most vascular beds (27), which may arise from the activity of the local myogenic mechanism (28).

Intravital microscopy (IVM), laser Doppler flowmetry, and blood oxygen level–dependent (BOLD) can be used to detect fluctuations in blood flow (29–31). The fluctuations can be transformed into frequency domain by either Fourier or wavelet analysis to analyze the fluctuation power (32).

Deoxyhaemoglobin content time series in blood flow could be easily detected by BOLD with high temporal and spatial resolution (33–35). Deoxyhaemoglobin has the T2* effect. The spatial and temporal heterogeneity of spontaneous T2* MR signal fluctuations was first observed in an implanted fibrosarcoma mouse model by Baudelet et al. (36, 37) and later confirmed in tumor xenograft models of colorectal carcinoma by Gonçalves and colleagues (38). However, the occurrence of spontaneous BOLD signal fluctuations has not been well demonstrated in human MSK tumors except in our previous published work (39).

In our previous work, we extracted BOLD signal fluctuations of the 48 MSK tumors by manually selecting the ROIs (regions of interest) in the peripheral and central regions of tumor. The results showed that mean power (the “power” is the square of the BOLD fluctuation amplitude with arbitrary unit) of BOLD fluctuation at a frequency band of 0.073–0.198 Hz was stronger in the peripheral than central regions of the malignant tumors (P < 0.05), whereas no such difference for the benign tumors were there (39), which may be due to the vasculature difference between the malignant and benign MSK tumors. However, it still did not allow us to differentiate malignant from benign MSK tumors just according to this feature (39).

In the current study, we hypothesized that benign MSK tumors have higher fluctuation power of the BOLD signal on some specific frequency than malignant tumors, specifically focused on the frequency band of 0.073–0.198 Hz. MSK tumors were analyzed by using independent component analysis (ICA) that is a method of blind source separation without defining ROI. ICA can decompose the observed data into statistically independent components (40), and it is capable of extracting desired tumor BOLD fluctuation signal from noise (e.g., scanner, physiological, and motion artifacts). After that, we analyzed the tumor BOLD fluctuations *via* power spectrum analyses on 96 specific frequency points ranging from 0 to 0.25 Hz especially in the band of 0.073–0.198 Hz to see if any fluctuation power can be used to differentiate benign from malignant MSK tumor.

## Materials and methods

### Participants

Ninety-two patients (58 men and 34 women; age, 37.4 ± 18.2 years, 11–73 years) who were hospitalized from March 2009 to May 2014 with primary MSK tumors were included in this study. This sample size included 43 cases from our previous study (28 men and 15 women; age, 36.3 ± 17.6 years, 14–73 years). The final diagnosis of the tumors was determined by histological testing after resection or needle biopsy. All the patients were drug-naive and had no treatment prior to MRI. The inclusion criteria were as follows: 1) the tumor had to be predominately solid; 2) the tumor dimension had to be larger than 3 cm in each of the axial, sagittal, and coronal plains; and 3) the tumor had no extensive intratumor necrosis or bleeding. This study was approved by the Institutional Research Ethics Board in the Third Hospital of Hebei Medical University, China, and all patients signed a written informed consent before the study was carried out.

### Magnetic resonance imaging

**Figure 1:**
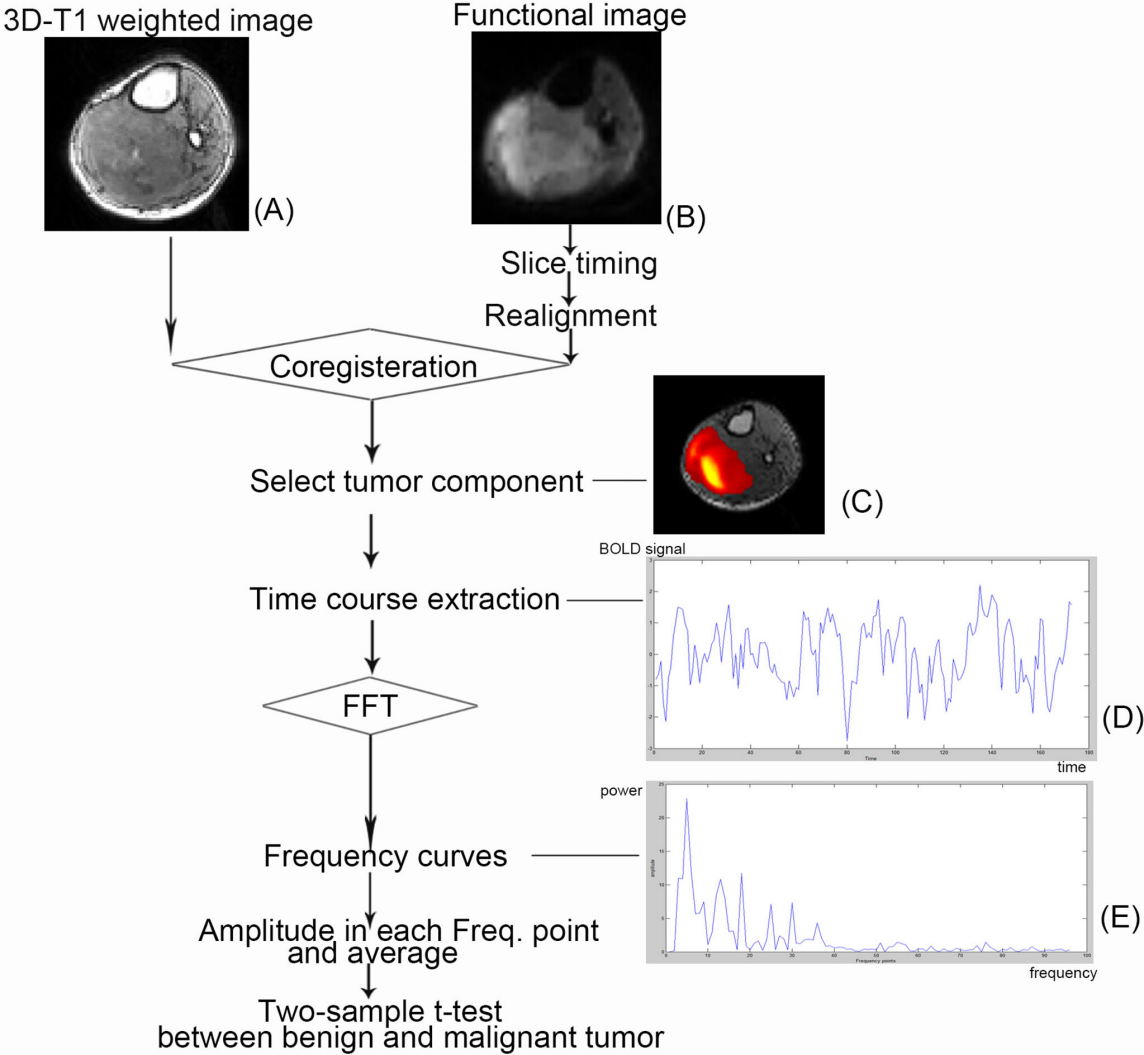
Schematic illustration of the course to data processing and analysis. The selected patient is a 57-year-old man with primitive neuroectodermal tumor in his right calf. **(A)** A functional image. **(B)** A 3D T1-weighted image. **(C)** The tumor component selected in independent component analysis. **(D)** The time course after preprocessing and independent component analysis. **(E)** Power spectrum using fast Fourier transformation (FFT).

MRI was performed on a 1.5-T Siemens MR scanning system (Avanto, Siemens, Erlangen, Germany). Patients were imaged in the supine position and were asked to relax. Depending on which body part was under study, image acquisition used one of the following coils: a large body matrix coil, an eight-channel knee coil, or an eight-channel body array coil. T1-weighted imaging (3D T1WI) is based on the three-dimensional turbo fast low-angled shot (**Figure 1A**) (repetition time/echo time, 1,900/2.97 ms; flip angle, 15°; number of slices, 176; slice thickness/gap, 1/0.5 mm; acquisition matrix, 256 × 246; field of view, 220 × 220 mm), and BOLD fMRI is based on the two-dimensional echo planar imaging (**Figure 1B**) with 20 axial slices (repetition time/echo time, 2,000/40 ms; slice thickness/gap, 5/1 mm; field of view, 220 × 220 mm; acquisition matrix, 64 × 64; voxel size, 3.44 × 3.44 × 6.0 mm; dummy scans, 3; scanning time, 6 min, total of 177 scans).

### Data preprocessing

**Figure 2:**
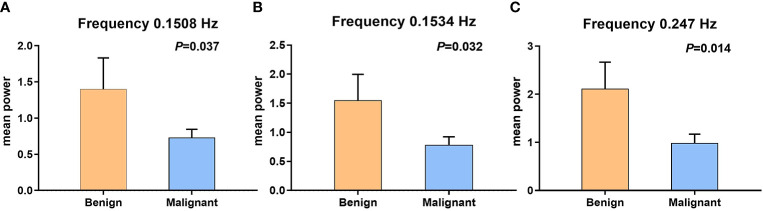
The error bars of two sample *t*-test between benign and malignant tumors, which have statistical significance. **(A)** Fifty-eighth frequency point (0.1508 Hz); *P*-value is 0.037. **(B)** Fifty-ninth frequency point (0.1534 Hz); *P*-value is 0.032. **(C)** Ninety-fifth frequency point (0.247 Hz); *P*-value is 0.014.

Preprocessing and analysis of fMRI data were carried out using DPARSFA v2.3 (41) and REST v1.8 (42) based on SPM8 (https://www.fil.ion.ucl.ac.uk/spm) and MATLAB 2012a (the MathWorks, Inc., Natick, MA). **Figure 2** shows the flowchart of the whole analysis procedure. The following prestatistics processing was applied: 1) the fMRI data was converted to Neuroimaging Informatics Technology Initiative format; 2) the first four frames (i.e., data acquired in first 8 s) were discarded in consideration of machine equilibrium and subjects’ adaptation to the scanning, leaving 173 frames for further analysis; 3) slice timing correction was applied to correct within-scan acquisition time differences between slices by using sinc interpolation; 4) patient’s body motion was corrected using a six-parameter rigid-body transformation, and the patients that were found to have excessive body motion (>3 mm or >3°) were excluded from further analysis; 5) the 3D high-resolution T1 image of each patient was co-registered to their own averaged BOLD fMRI image to spatially match the two modalities; and 6) finally, the images were smoothed using a 6-mm full-width-at-half-maximum isotropic Gaussian kernel to improve signal-to-noise ratio (SNR).

### Independent component analysis

After the preprocessing steps mentioned above, individual-level ICA, a data-driven method, was used to identify the component related to tumor tissue from resting-state fMRI (rs-fMRI) data for each patient. With optimized total component number (i.e., model order) set to 10, individual-level ICA was applied to the preprocessed rs-fMRI data using MICA toolbox (22). In MICA, each subject’s preprocessed rs-fMRI data were fed into a one-stage principal component analysis to perform data dimension reduction temporally. After that, ICA decomposition was performed by using an Infomax algorithm. Such an analysis was performed for 100 times, each time with randomized initial value to produce consistent and reliable ICA decompositions (43, 44). Individual subject components were derived from back-reconstruction and z-score transformation. After that, the software (MICA toolbox) took the 10 signal fluctuation components in the form of color pictures. Then, that would be matched to its T1WI image. The color pictures that can basically cover the T1WI images of the tumor were considered to represent the frequency signal of tumor (tumor-related component). This work was done by two experts engaged in medical imaging and data analysis (Lisha Duan and Huiyuan Huang) (**Figure 1C**).

### Power spectrum analyses

For each patient in two groups, the tumor-related BOLD time series was extracted and transformed from temporal to frequency domain with a fast Fourier transformation using MATLAB (**Figures 1D, E**) (i.e., frequency power spectrum, where the “power” is the square of the BOLD fluctuation amplitude). Because there were 173 time points in the temporal domain, the power spectrum finally accounted for 96 frequency points.

### Statistical analyses

A two-sample *t*-test was used to assess whether there was a significant difference in the fluctuation powers at each frequency point (total of 96) between benign and malignant MSK tumors. P < 0.05 was considered to indicate a significant difference.

### Receiver *operator characteristic analyses*

The difference of the powers at each frequency point between the benign and malignant MSK tumors was tested by using two-sample *t*-test, and then, the receiver operator characteristic (ROC) analysis was performed on SPSS v13.0 software (SPSS Inc., Chicago, IL, USA) to assess the diagnostic capability of distinguish between benign and malignant MSK tumors.

## Results

### Participants and histopathology

Of the 92 MSK tumor patients, four cases with excessive body motions (translation > 3 mm or rotation > 3°) were excluded for further study. Fourteen cases after ICA were not found clear tumor-related component and thus were removed from further analyses. The failed cases included nine cases from our previous study (five malignant tumors and four benign tumors). The data from the remained 74 patients were put into further analyses (including 34 cases from previous study; six benign tumors and 28 malignant tumors) (**Table 1**). Histological results showed that 56 cases were malignant tumors and 18 cases were benign tumors.

**Table 1:** Demographic and tumor information of 74 cases with malignant or benign musculoskeletal tumors.

Variables (malignant)^a^	Values	Variables (benign)^b^	Values
**Cases**	56	**Cases**	18
**Age (yrs)**		**Age (yrs)**	
Mean ± std	37.39 ± 19.89	Mean ± std	36.71 ± 13.34
Range	11–73	Range	14–57
**Men-no. (%)**	45 (70.31)	**Men-no. (%)**	11 (45.83)
**Position-no. (%)**		**Position-no. (%)**	
Femur	25 (39.06)	Femur	6 (25.00)
Tibia	12 (18.75)	Tibia	8 (33.33)
Fibula	2 (3.12)	Fibula	2 (8.33)
Humerus	3 (4.69)	Humerus	3 (12.50)
Sacrum	1 (1.56)	Sacrum	n.s.
Os Innominatum	4 (6.25)	Os Innominatum	n.s.
Calcaneus	1 (1.56)	Calcaneus	n.s.
Soft tissue	16 (25.00)	Soft tissue	5 (20.83)
Tumor volume *(cm^3^)		Tumor volume *(cm^3^)	
Mean ± std	163.02 ± 140.88	Mean ± std	159.13 ± 180.96
Range	23.403–546.60	Range	19.60–588.60

^a^Osteosarcoma, malignant fibrous histiocytoma, synovial sarcoma, alveolar sarcoma, Ewing sarcoma, liposarcoma, chondrosarcoma, metastatic adenocarcinoma, primitive neuroectodermal tumor, malignant chondroblastoma, epidermoid leiomyosarcoma, fibrosarcoma, malignant tenosynovial giant cell tumor, lymphoma, malignant peripheral nerve sheath tumor, plasma cell tumors, mesenchymal malignant tumor, pleomorphic sarcoma, and pleomorphic rhabdomyosarcoma. ^b^Giant cell tumor of bone, neurofibroma, fibromatosis, giant cell tumor of bone associated with aneurysmal bone cyst, low-grade malignant fibrous histiocytoma, and atypical fibroblastic tumor. yrs, years; no, number. * Selected 37 tumors to calculate their volumes using formula: (4/3)πabc (a, b, c = the three radiuses). N.s., not specified.

### Statistical analyses

The BOLD fluctuation power of benign MSK tumors was significantly higher than that of malignant ones on the frequency points 0.1508 Hz (P = 0.037), 0.1534 Hz (P = 0.032), and 0.247 Hz (P = 0.014), respectively, by two-sample *t*-test analysis (**Figure 2** and **Table 2**).

**Table 2:** Three frequency points possess statistical significance in comparing the difference fluctuation power between the malignant and benign tumors in all frequency points (sum of 96 points).

Freq. points	Benign (N = 18)		Malignant (N = 56)		t-value	*P*-value
	*Mean*	*Std. Error*	*Mean*	*Std. Error*		
58th (0.1508 Hz)	1.400	0.4310	0.7330	0.1133	2.127	0.0369
59th (0.1534 Hz)	1.550	0.4473	0.7872	0.1374	2.106	0.0321
95th (0.247 Hz)	2.117	0.5484	0.9857	0.1867	2.510	0.0143

Two-sample t-test. The test standard is 0.05.

**Figure 3:**
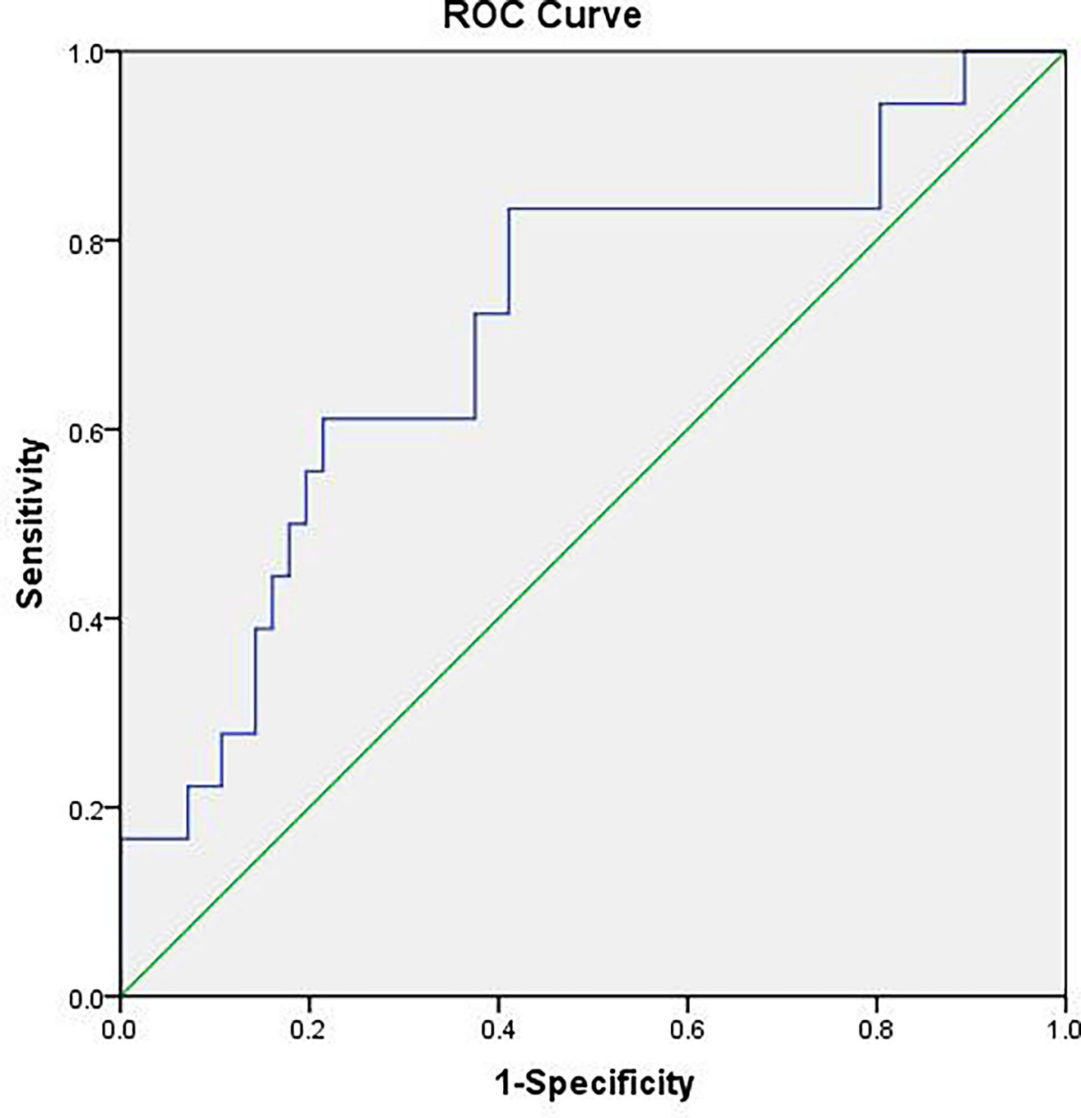
The receiver operating characteristic (ROC) curve of mean power of three frequency points (0.1508, 0.1534, and 0.247 Hz). The area under the ROC curve value was 0.706 (P = 0.009). The mean power of musculoskeletal tumor is 0.73130, which means that, if the power value is greater than or equal to 0.73130, then it is considered benign MSK tumor with sensitivity of 83% and specificity of 59%.

The difference of the powers at each frequency point between the benign and malignant MSK tumors was tested by using two-sample *t*-test, and then, the ROC analysis was used to assess the diagnostic capability of distinguish between benign and malignant MSK tumors. The ROC analysis of mean power of three frequency points (0.1508, 0.1534, and 0.247 Hz) showed that the area of under ROC curve is 0.706 [P = 0.009; 95% confidence interval (CI), 0.563–0.850], and the cutoff value was 0.73130. If the benign MSK tumor was diagnosed with the mean power value greater than or equal to the cutoff value of 0.73130, then the sensitivity and the specificity were 83% and 59%, respectively (**Figure 3**).

**Figure 4:**
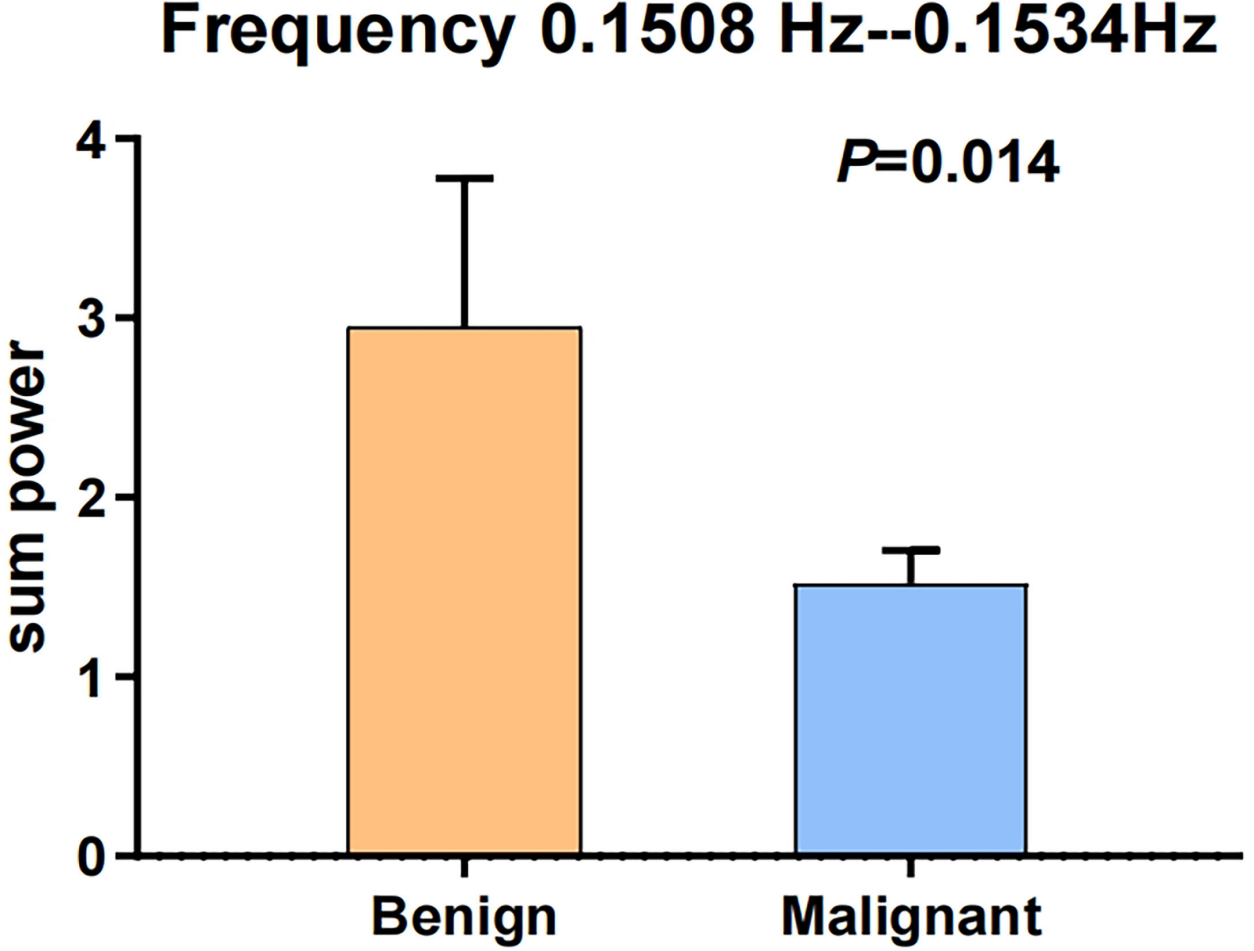
The error bars of two sample *t*-test after merging the two frequency points of 0.1508 and 0.1534 Hz. The sum power of benign MSK tumors was significantly higher than that of malignant tumors. *P*-value is 0.014.

**Figure 5:**
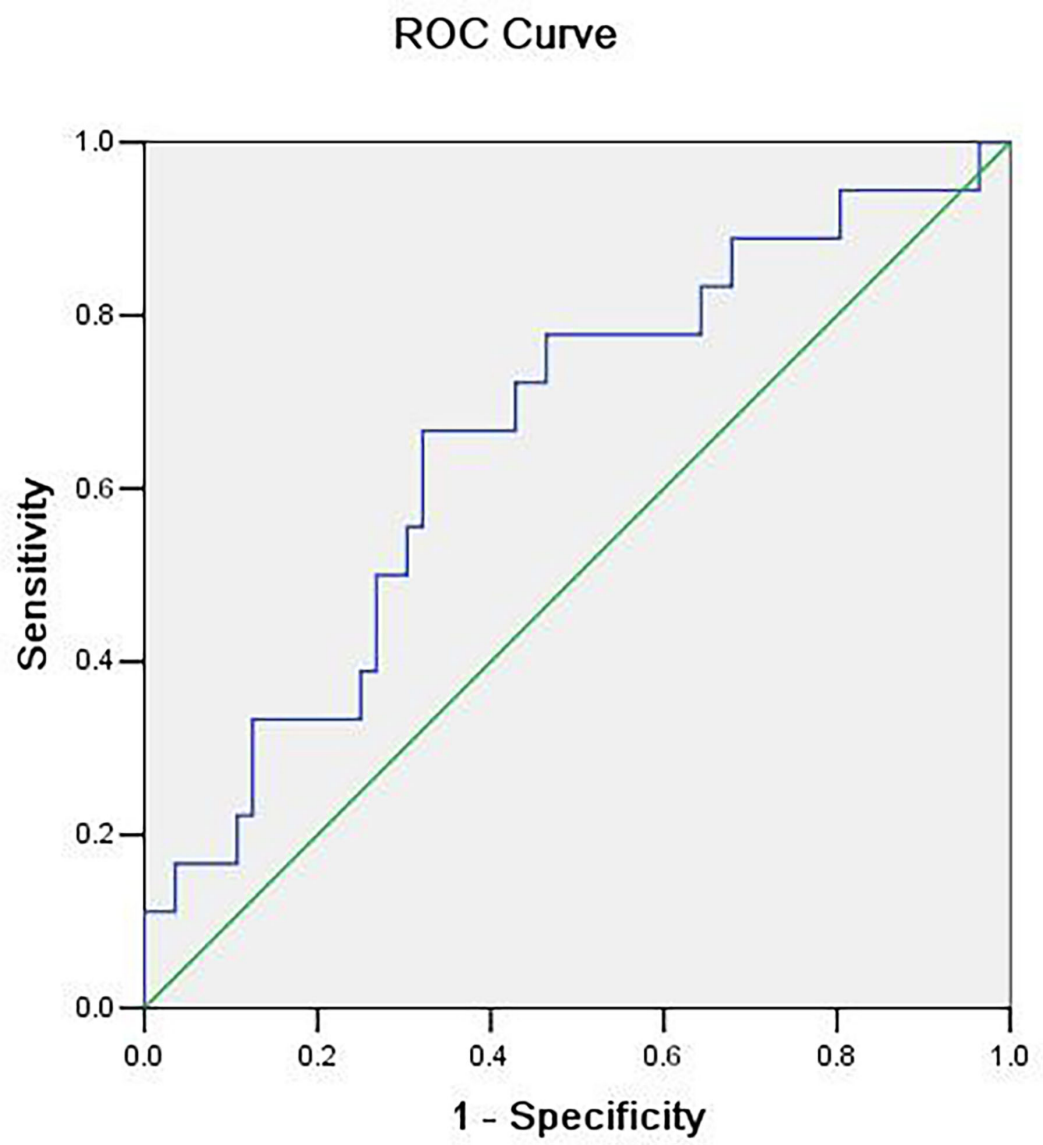
The receiver operating characteristic (ROC) curve of sum power of two frequency points (0.1508 and 0.1534 Hz). The area under the ROC curve value was 0.661 (P = 0.041). The cutoff value is 1.41241, which means that, if the sum power value is greater than or equal to 1.41241, then it is considered benign MSK tumor with sensitivity of 67% and specificity of 68%.

These data were further processed with *post hoc* analysis. The two frequency points of 0.1508 and 0.1534 Hz may be related to the frequency of myogenic activity in the arteriole (45–49). Hence, we further merged the two frequency points of 0.1508 and 0.1534 Hz to compare the sum powers between benign and malignant MSK tumors. The difference of the sum powers between the benign and malignant MSK tumors was tested by using two-sample *t*-test. The result showed that the sum powers of benign MSK tumors was significantly higher than that of malignant ones (P = 0.014) (**Figure 4**). The ROC analysis of sum powers of two frequency points (0.1508 and 0.1534 Hz) showed that the area of under ROC curve is 0.661 (P = 0.041; 95% CI, 0.515–0.806), and the cutoff value was 1.41241. If the benign MSK tumor was diagnosed with the sum power value greater than or equal to the cutoff value of 1.41241, then the sensitivity and the specificity were 67% and 68%, respectively (**Figure 5**).

## Discussion

In our previous study, we extracted BOLD signal fluctuations from different regions of the MSK tumor by manually selecting the ROIs, and we characterized them in benign and malignant MSK tumors *via* power spectrum analyses in pre-established low-frequency bands (band-1, 0.01–0.027 Hz; band-2, 0.027–0.073 Hz; band-3, 0.073–0.198 Hz; and band-4, 0.198–0.25 Hz). It was found that BOLD fluctuations at 0.073–0.198 Hz were stronger in the peripheral than central regions of the malignant tumors, and no such difference was observed for the benign tumors (13 benign tumors and 35 malignant tumors) (39).

In current study, the integral MSK tumor-related signal components were extracted by using ICA without defining ROI. After that, we analyzed the powers *via* power spectrum analyses on 96 specific frequency points ranging from 0 to 0.25 HZ. The result showed that there was significant difference in power at the frequency points of 0.1508 Hz (P = 0.037), 0.1534 Hz (P = 0.032), and 0.247 Hz (P = 0.014), respectively, between the benign and malignant MSK tumors after two-sample *t*-test analysis (**Table 2**).

BOLD signal usually is very weak and can easily be masked by noise. Although we used the two different signal extraction methods and data analysis methods in our two studies, the significant fluctuation power of two frequency points of 0.1508 Hz (P = 0.037) and 0.1534 Hz (P = 0.032) between the benign and malignant MSK tumors in this study is consistent with that of the significant frequency band of 0.073–0.198 Hz (P < 0.05) in the previous study, which may indicate that the significant fluctuation power around 0.073–0.198Hz is really true.

ICA is a method of blind source separation, which linearly decomposes the observed data into statistically independent components and separates noise and artifacts effectively, making the tumor detection robust to imaging noise (50, 51). It is a multivariate signal processing method to explore the spatiotemporal properties of fMRI data (52–54). The test–retest reliability of ICA on functional component detection generally stays high (55, 56). In addition, previous BOLD fMRI studies have demonstrated the feasibility of rs-fMRI-based automatic tumor tissue identification by using individual ICA (57, 58).

The ICA method is better than the defined ROI method used by previous study, because ICA is capable of extracting desired tumor BOLD time series from noise (e.g., scanner, physiological, and motion artifacts). Moreover, the variability caused by the operator-defined ROI can be avoided by using the ICA method. In particular, the great heterogeneity in tumors may also mask the really signal by using defined ROI method.

Interestingly, the frequency band of 0.073–0.198 Hz in tumors has been suggested to be caused primarily by vascular myogenic activity in vasomotion (45–49). Vasomotion is a phenomenon of blood flow in normal tissue, which *in vivo* is associated with the rhythmic oscillations in vessel diameter (26, 59). These oscillations of the lumen diameter modify blood flow in a corresponding fashion and produce periodical fluctuations known as flowmotion (26, 60).

The frequency of myogenic activity in the arteriole (0.052–0.15Hz) is similar to the first two frequency points (0.1508 and 0.1534 Hz) related to MSK tumors in current study and the band of 0.073–0.198Hz in the previous study, which may indicate that different fluctuation power of frequency points between the malignant and benign MSK tumors is related to the different power of vasomotion between the malignant and benign MSK tumors (39).

The BOLD fluctuation power of benign MSK tumors was significantly higher than that of malignant ones on three frequency points. The difference in the power of the benign and malignant tumors may be explained by the differences of their blood vessel structure (24, 61). The blood vessels, especially smooth muscle cells, are relatively mature in benign tumor compared with the malignant one (21). Relative normal function of smooth muscle may explain higher power of frequency points (0.1508 and 0.1534 Hz) that indicate vasomotion status.

The ROC analyses of mean power of three frequency points (0.150, 0.1534, and 0.247 Hz) showed that the area under the ROC curve value was 0.706 (P = 0.009; 95% CI, 0.563–0.850), and the cutoff value was 0.73130, which means that, if the mean power value is greater than or equal to 0.73130, then it is considered benign MSK tumor, and the sensitivity and specificity are 83% and 59%, respectively. The AUC was 0.706, whose diagnosis accuracy is moderate. This indicates that the mean power of BOLD fluctuation at the frequency points of 0.1508, 0.1534, and 0.247 Hz, as a biomarker can be used to differentiate the benign from malignant MSK tumors with a high sensitivity but low specificity (**Figure 4**). Our current results are similar to new developed state-of-the-art methods such as DWI, PWI, and MRS for differentiating benign from malignant MSK tumors with low specificity (15–18).

The ROC analyses of sum power of two frequency points (0.1508 and 0.1534 Hz) showed that the cutoff value was 1.41241. If the benign MSK tumor was diagnosed with the sum power value greater than or equal to the cutoff value of 1.41241, then the sensitivity and the specificity were 67% and 68%, respectively. This specificity is higher than that analyzed by mean power of three frequency points (0.1508, 0.1534, and 0.247 Hz) above. It indicated that the frequency points of 0.1508 and 0.1534 Hz could better reflect the true difference between benign and malignant MSK tumors.

The ICA method used to detect tumor-related signal component has the potential to determine the boundary of the tumor more precisely and delineate the tumor tissue from the surrounding healthy tissues (57), which may be helpful for making a comprehensive presurgical planning, especially for tumor resection or other image-directed interventions.

The relatively high frequency (0.247 Hz, P = 0.014), whose pathophysiological significance we have not yet understood, may be a noise because of the many factors that affect BOLD imaging. Perhaps, it represents a different kind of pathophysiology, which needs to be explored.

This study had limitations. First, the substantially smaller sample size of benign tumors compared with malignant tumors may induce statistical bias. Second, the sensitivity (83%) and the specificity (59%) are low when our new biomarker is used to differentiate benign from malignant MSK tumors.

In the future, more research studies should be done to improving its sensitivity and specificity. For example, the BOLD signals unrelated to the MSK tumor should be further removed through advanced algorithm. In particular, the more precise methods to acquire the powers of BOLD fluctuations at the frequency points of 0.1508, 0.1534, and 0.247 Hz are needed, of which the first two frequency points may be related to vascular myogenic activity. The sensitivity and the specificity of other functional MRI techniques (PWI, MRS, and DWI) (62–66) (**Table 3**) in the literature were low too for differentiating benign from malignant MSK tumors. The research studies should be conducted to clarify if the sensitivity and specificity can be promoted when this new biomarker is associated with other biomarkers.

**Table 3:** Advanced functional MRI techniques (PWI, MRS, and DWI) to differentiate benign and malignant musculoskeletal tumor and their sensitivities and specificities.

Advanced technique	Parameter	P-value	Cutoff value	Diagnostic performance		
				Accuracy (%)	Sensitivity (%)	Specificity (%)
PWI	Ktrans (62)	p = 0.028	0.19 min^−1^	–	79^☆^	27^☆^
	Ve (62)	NS	0.37min^−1^	–	79^☆☆^	38^☆☆^
	FP Slope (63)	P < 0.001	45%/s	76	74	77
MRS	Choline SNR (64)	P < 0.001	–	73.3	–	–
	PoA of cho (18)	NS	Presence/absence	–	50^☆☆^ 82.4^☆^	61.5^☆☆^ 64.3^☆^
DWI	ADC* (65)	P < 0.01	1.45 × 10^−3^ mm^2^/s	–	90.9	60
	ADC** (66)	P = 0.004	1.132 × 10^−9^ mm^2^/s	–	83	81

Perfusion-weighted imaging (PWI), magnetic resonance spectroscopy (MRS), diffusion-weighted imaging (DWI), the transfer rate of plasma contrast agent to the extracellular extra vascular space (Ktrans), the volume fraction of the extracellular extra vascular space (Ve), first pass (FP), signal-to-noise ratio (SNR), the presence or absence of a choline peak at 3.2 ppm (PoA of cho), apparent diffusion coefficient (ADC), soft tissue tumors (STT), and not statistically significant (NS). *Non-myxoid STT; **non-myxoid, nonhaemosiderin STT; ☆STT; ☆☆ bone tumor.

In conclusion, we found that the BOLD fluctuation power of the benign tumors is higher than that of malignant MSK tumors on the frequency points of 0.1508, 0.1534, and 0.247 Hz using the ICA method to extract tumor BOLD time series. By combining the power of 0.1508 and 0.1534 Hz, we could better detect the difference between benign and malignant MSK tumors with higher specificity. The meaning of this difference remains under investigation. However, our finding suggested that BOLD fMRI allows a non-invasive *in vivo* study that avoids contrast agent administration, with a potential to differentiate between malignant and benign MSK tumors. Furthermore, this result was more effective and clear to distinguish between benign and malignant MSK tumors than our previous study.

## Data availability statement

The raw data supporting the conclusions of this article will be made available by the authors, without undue reservation.

## Ethics statement

The studies involving human participants were reviewed and approved by Ethics Committee of the Third Hospital of Hebei Medical University. Written informed consent to participate in this study was provided by the participants’ legal guardian/next of kin.

## Author contributions

LD, JC, and HZ contributed to conception and design of the study. LD wrote the first draft of the manuscript. HH wrote sections of the manuscript. LD, FS, ZZ, MW, and MX collected the required MRI data. XX provided and analyzed the pathological data required for the study. LD collected and assembled the total data. LD, HZ, HH, YZ, MW and HY performed the statistical analysis and interpretation. LD and JC determined the selection of references and experimental standards and performed the MRI data analysis and interpretation. JC and YZ performed manuscript approval and modifification. All authors contributed to manuscript revision, read, and approved the submitted version.

## Funding

This work was supported by the Medical Science Research Foundation of Hebei Province (No.16277706D) and partially supported by the Key-Area Research and Development Program of Guangdong Province (No. 2021B0101420006), Shanghai Pilot Program for Basic Research-Chinese Academy of Science, Shanghai Branch (No. JCYJ-SHFY-2022-014), the China Postdoctoral Science Foundation (No. 2020M682682), Guangdong Province Philosophy and Social Science Foundation for Youths (No. GD21YXL02), Basic and Applied Basic Research Project of Guangzhou Basic Research Program (No. 202201011471).

## Conflict of interest

The authors declare that the research was conducted in the absence of any commercial or financial relationships that could be construed as a potential conflict of interest.

## Publisher’s note

All claims expressed in this article are solely those of the authors and do not necessarily represent those of their affiliated organizations, or those of the publisher, the editors and the reviewers. Any product that may be evaluated in this article, or claim that may be made by its manufacturer, is not guaranteed or endorsed by the publisher.
